# Young Aggressive Prostate Tumors Exhibit Increased Genomic Instability and an Exploratory RNA–CNA-Concordant Transcriptomic Pattern

**DOI:** 10.3390/ijms27188316

**Published:** 2026-09-18

**Authors:** Fernando Bergez-Hernández, Alejandra Martínez-Camberos, Geovanni Romero-Quintana, Jesús Parra-Unda, Lennin Garrido-Palazuelos, Martín Irigoyen Arredondo

**Affiliations:** 1 Unidad Regional Mazatlán, Laboratorio de Investigación Biomédica y Biotecnológica, Universidad Autónoma de Occidente, Av. del Mar 1200, Col. Tellería, Mazatlán C.P. 82100, Sinaloa, Mexico; fernando.bergez@uadeo.mx (F.B.-H.); alejandra.martinez@uadeo.mx (A.M.-C.); 2 Facultad de Ciencias Químico-Biológicas, Universidad Autónoma de Sinaloa, Ciudad Universitaria, Calz. de las Américas Nte. 2771, Col. Burócrata, Culiacán Rosales C.P. 80030, Sinaloa, Mexico; geovanniromero@uas.edu.mx; 3 Unidad de Investigaciones en Salud Pública “Dra. Kaethe Willms”, Facultad de Ciencias Químico-Biológicas, Universidad Autónoma de Sinaloa, Ciudad Universitaria, Culiacán Rosales C.P. 80013, Sinaloa, Mexico; ricardoparraund@uas.edu.mx; 4 Unidad Regional Los Mochis, Departamento Académico de Ciencias de la Salud, Universidad Autónoma de Occidente, Blvd. Macario Gaxiola y Carretera Internacional México 15, Los Mochis C.P. 81223, Sinaloa, Mexico; lennin.garrido@uadeo.mx

**Keywords:** young-onset prostate cancer, transcriptomic alterations, genomic instability, copy-number alterations, multi-omics integration

## Abstract

Prostate cancer (PCa) is a highly heterogeneous disease, and the molecular features underlying differences in tumor aggressiveness remain incompletely understood. This study aimed to characterize the molecular differences between young aggressive (YA) and old indolence-associated (OI) prostate tumors using an integrative multi-omics approach. RNA sequencing, copy number alteration (CNA), and clinical data from The Cancer Genome Atlas Prostate Adenocarcinoma (TCGA-PRAD) cohort were analyzed. YA tumors (age ≤ 58 years, Gleason score ≥ 8; n = 30) were compared with OI tumors (age ≥ 67 years, Gleason score ≤ 7; n = 41). YA tumors exhibited higher FGA and a greater nominal frequency of homozygous PTEN deletion, consistent with increased genomic instability. Transcriptomic analysis identified 1083 differentially expressed genes, whereas integrative RNA–CNA analysis prioritized 40 exploratory concordant genes, 35 of which showed copy-number loss-associated downregulation. Sensitivity analyses showed that 8 of 40 genes retained both the predefined FDR and effect-size criteria after tumor-purity adjustment, whereas only one gene retained both criteria after additional adjustment for pT and N. Functional enrichment and network analyses identified metabolic and homeostatic programs and a central interaction module within the original prioritized gene set. These findings provide exploratory hypotheses regarding molecular differences between the two predefined extreme phenotypes.

## 1. Introduction

Prostate cancer (PCa) is the second most frequently diagnosed malignancy among men and remains one of the leading causes of cancer-related mortality worldwide [1]. Despite substantial advances in diagnosis and treatment, disease progression remains highly heterogeneous, largely reflecting the complex genomic landscape of PCa. Tumor evolution is driven by the accumulation of diverse molecular alterations, including chromosomal gains and losses, copy number alterations (CNAs), gene fusions, somatic mutations, and epigenetic changes that collectively shape tumor behavior and therapeutic response [2]. Recurrent chromosomal deletions involving tumor suppressor genes such as NKX3.1, *PTEN*, RB1, and *TP53*, together with alterations affecting androgen receptor (AR), DNA damage repair (DDR), and PI3K/AKT signaling pathways, represent major drivers of prostate carcinogenesis and progression [2,3].

Genomic instability has emerged as a hallmark of aggressive PCa and an important determinant of clinical outcome [4]. Comprehensive genomic profiling has demonstrated that CNAs, chromosomal aneuploidy, and pathogenic genomic variants are associated with adverse clinicopathological features, disease progression, and reduced survival [2,5]. Beyond their prognostic significance, these genomic alterations have become increasingly relevant for patient stratification and the implementation of pathway-directed targeted therapies [6].

Although PCa predominantly affects older men, approximately 10% of cases are diagnosed in individuals aged 55 years or younger [7,8]. The incidence of early-onset PCa has increased over recent decades, partly following the introduction of prostate-specific antigen (PSA) screening [8,9]. This distinction is reinforced by the stronger hereditary component consistently reported in younger patients and the remarkable clinicopathological differences [10]. Previous studies have reported that PCa diagnosed at younger ages may exhibit distinct clinicopathological and hereditary characteristics, with aggressive features observed in a subset of younger patients [7,9,10]. Consequently, genetic risk prediction may have greater clinical utility in younger men than in older populations [8].

Despite this evidence, the molecular features distinguishing the YA extreme phenotype and the OI phenotype remain incompletely characterized, particularly regarding the relationship between genomic alterations and transcriptional regulation. It remains unclear whether these tumors are driven primarily by canonical AR signaling, genomic instability, CNA-associated transcriptional patterns, or distinct regulatory programs involving non-coding RNAs.

In the present study, we performed an integrative multi-omics analysis of The Cancer Genome Atlas Prostate Adenocarcinoma (TCGA-PRAD) cohort to characterize the molecular features associated with the predefined YA phenotype. By comparing extreme clinical phenotypes defined as young aggressive (YA) and old indolence-associated (OI) tumors, we sought to identify the genomic and transcriptomic features that distinguish these two clinically contrasting extreme phenotypes. Our findings reveal that YA tumors are characterized by increased chromosomal instability, recurrent *PTEN* loss, distinct miRNA regulatory patterns, and widespread RNA–CNA concordant transcriptional changes, supporting genomic instability as a prominent molecular feature of the YA extreme phenotype.

## 2. Results

### 2.1. Clinical Stratification Identifies Biologically Distinct Prostate Cancer Phenotypes

To investigate the molecular features associated with the predefined YA phenotype, we first defined two extreme clinical phenotypes within the TCGA-PRAD cohort to maximize biological contrast, RNA-seq data from TCGA-PRAD initially included 554 samples, of which 501 corresponded to primary tumors. Among these, 283 tumors contained complete age and pathological Gleason score metadata suitable for phenotype stratification. Applying strict age and Gleason-based selection criteria yielded a final cohort of 71 tumors, comprising 30 YA tumors and 41 OI tumors. YA tumors were defined as patients aged ≤ 58 years with Gleason score ≥ 8, whereas OI tumors were defined as patients aged ≥ 67 years with Gleason score ≤ 7. Within the YA cohort, 11 tumors were classified as Gleason 8 and 19 as Gleason 9. In contrast, the OI group included 8 tumors with Gleason 6 and 33 tumors with Gleason 7. This extreme phenotype design established two clinically contrasting groups representing the YA and OI phenotypes. To further assess the potential contribution of age within comparable tumor-grade strata, the 283 evaluable tumors were also examined according to age and Gleason strata. The resulting groups comprised 82 younger and 41 older tumors within the Gleason ≤ 7 stratum, and 30 younger and 18 older tumors within the Gleason ≥ 8 stratum. These age-stratified groups were used for exploratory sensitivity analyses of the prioritized genes and PTEN CNA status (Supplementary Table S1).

### 2.2. Genomic Instability and Tumor Burden Differ Between YA and OI Tumors

**Figure 1:**
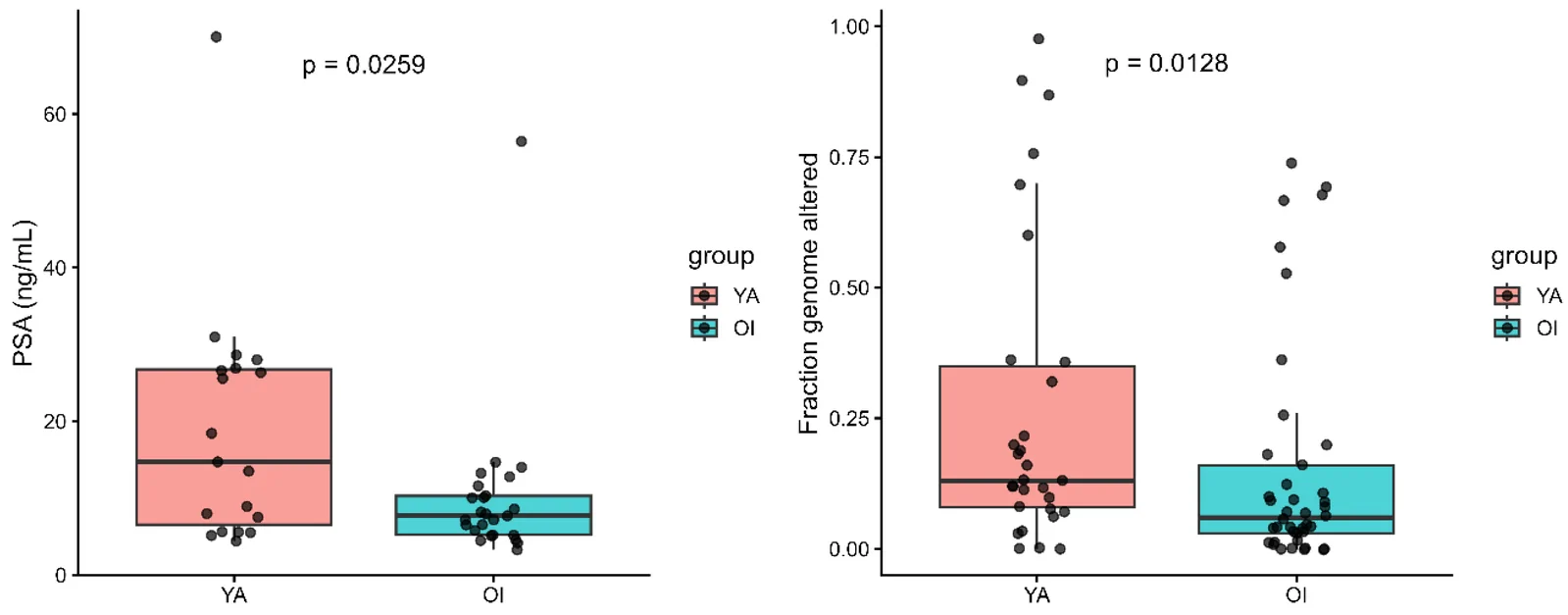
Clinical and genomic differences between YA and OI prostate tumors. Boxplots comparing preoperative prostate-specific antigen (PSA) levels and fraction genome altered (FGA) between YA (n = 30) and OI (n = 41) tumors. Each point represents an individual sample. Boxes indicate interquartile range (IQR), center lines represent medians, and whiskers extend to 1.5× IQR. Statistical comparisons were performed using the Wilcoxon rank-sum test. *p* values shown in the figure are nominal *p* values; for the prespecified clinical–pathological comparison family, statistical significance was assessed using Benjamini–Hochberg-adjusted *p* values. YA: Young aggressive; OI: Old indolence-associated.

We next evaluated whether YA and OI tumors differed in major clinical and genomic features associated with PCa aggressiveness. Preoperative PSA levels were higher in YA tumors compared with OI tumors (nominal *p* = 0.0259), although this difference did not remain statistically significant after Benjamini–Hochberg correction (FDR = 0.0725). Median PSA levels were substantially higher in YA tumors (14.7 ng/mL) compared with OI tumors (7.7 ng/mL). To assess genomic instability, we compared FGA, mutation burden, and absolute ploidy between groups. YA tumors exhibited higher FGA values than OI tumors (Wilcoxon rank-sum test, *p* = 0.0128), supporting increased chromosomal instability in YA extreme phenotype tumors (Figure 1). Median FGA values were 0.13 in YA tumors and 0.06 in OI tumors. In contrast, no significant differences were observed for mutation burden (*p* = 0.1227) or absolute ploidy (*p* = 0.7692). These findings suggest that the YA phenotype is characterized primarily by increased chromosomal instability rather than elevated mutational burden or major ploidy alterations. Prior treatment was reported in 2 OI tumors (4.9%) and none of the YA tumors (0%), with no evidence of a between-group difference (Fisher’s exact test, *p* = 0.508; FDR-adjusted *p* = 0.508). In the available TCGA clinical metadata, neoadjuvant treatment was recorded as “No” for all 71 tumors included in the primary analysis (100% completeness). However, the available treatment metadata do not comprehensively capture all forms of prior systemic therapy and therefore do not allow previous androgen-deprivation therapy or other systemic treatment to be excluded with certainty.

A comprehensive comparison of demographic, clinicopathological, and tumor-related characteristics is provided in Supplementary Table S2. Age and Gleason score are presented descriptively because they were used to define the study groups. For all other variables, effect estimates with 95% confidence intervals, nominal and Benjamini–Hochberg-adjusted *p* values within prespecified comparison families, and missing data are reported.

Preoperative PSA levels were higher in YA tumors than in OI tumors (nominal *p* = 0.0259), but this difference did not remain statistically significant after Benjamini–Hochberg correction (FDR = 0.0725). PSA values were available for 19 YA and 25 OI tumors, with 27 values missing. YA tumors also showed a higher proportion of T3–T4 disease (86.2% vs. 60.5%; OR = 3.99, 95% CI 1.06–18.97; nominal *p* = 0.029), although this difference did not remain significant after multiple-testing correction (adjusted *p* = 0.101). Differences in nodal involvement and residual tumor status were likewise not significant after correction.

Tumor purity and fraction of genome altered were significantly higher in YA tumors after multiple-testing correction (tumor purity: median [IQR], 0.725 [0.228] vs. 0.550 [0.320], rank-biserial correlation = 0.354, 95% CI 0.080–0.603, FDR-adjusted *p* = 0.0256; fraction of genome altered: 0.13 [0.27] vs 0.06 [0.13], rank-biserial correlation = 0.348, 95% CI 0.081–0.591, FDR-adjusted *p* = 0.0256). Because tumor purity differed significantly between YA and OI tumors, and pathological stage showed clinically relevant between-group differences, we performed sensitivity analyses to assess the robustness of the prioritized transcriptomic findings to potential confounding. Differential expression was therefore evaluated using three progressively adjusted models incorporating tumor purity alone, tumor purity plus pathological T stage (pT), and tumor purity plus pT and nodal status (N).

### 2.3. miRNA Molecular Subtypes Distinguish YA and OI Tumors

**Figure 2:**
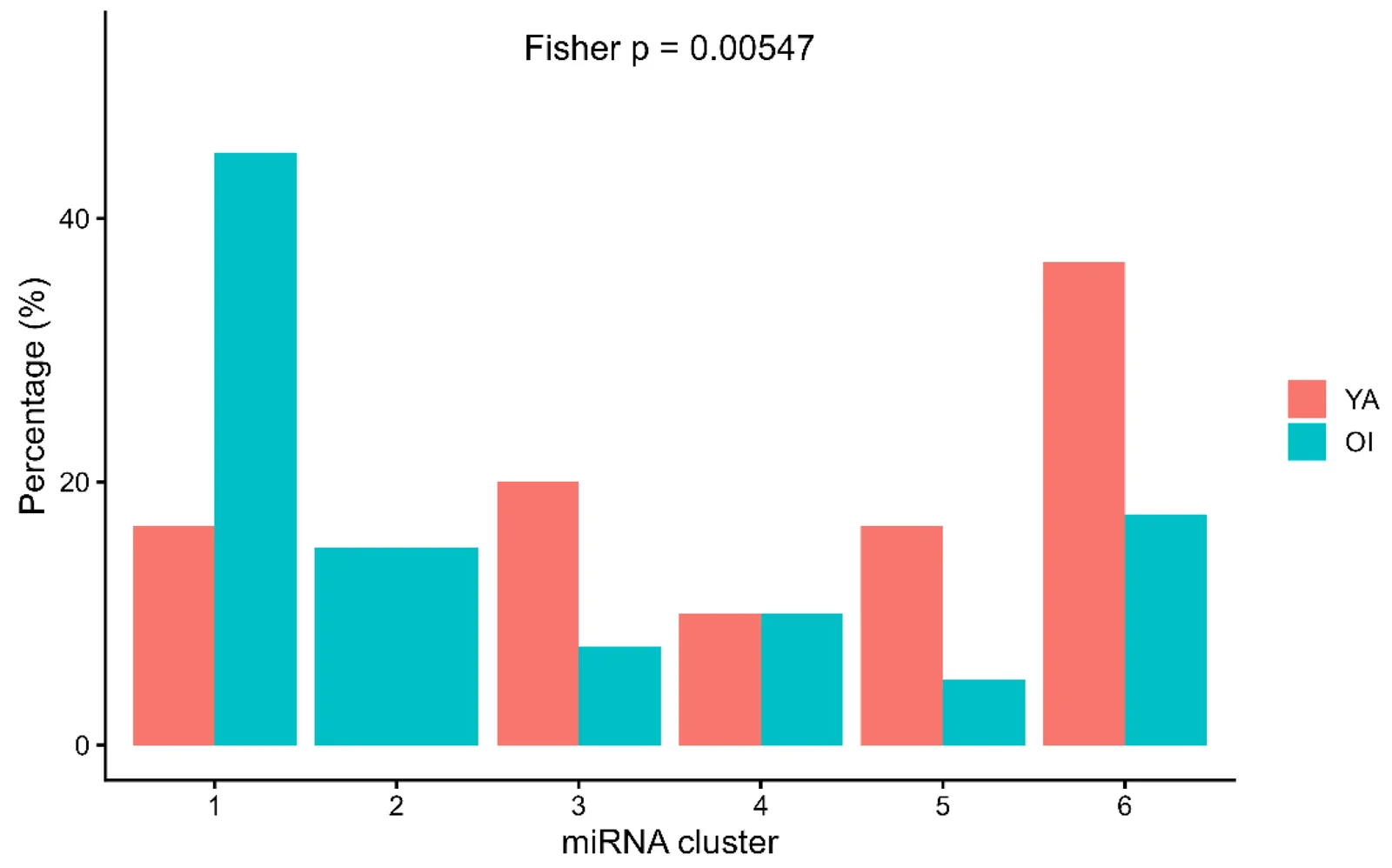
Differential distribution of miRNA molecular clusters between YA and OI tumors. Bar plots showing the relative distribution of TCGA miRNA molecular clusters in YA and OI prostate tumors. In Cluster 2, no YA tumors were represented, whereas OI tumors were distributed across the two clusters. Statistical significance was assessed using Fisher’s exact test. YA: Young aggressive; OI: Old indolence-associated.

To investigate whether YA and OI tumors differed in established molecular subtype classifications, we compared multiple TCGA-derived molecular subtype systems, including SCNA clusters, mRNA clusters, methylation clusters, and miRNA clusters. Among all subtype classification systems evaluated, only miRNA cluster distribution differed significantly between YA and OI tumors (Fisher’s exact test, *p* = 0.00547) (Figure 2).

No significant differences were observed for SCNA cluster (*p* = 0.2238), mRNA cluster (*p* = 0.0837), or methylation cluster (*p* = 0.2688). Detailed inspection of miRNA cluster distribution revealed marked differences between groups. OI tumors were enriched in miRNA cluster 1, whereas YA tumors showed relative enrichment in clusters 3, 5, and 6. These findings suggest that miRNA-mediated regulatory programs differ substantially between the YA extreme phenotype and the OI phenotype, implicating non-coding regulatory mechanisms in the YA phenotype.

### 2.4. PTEN Deletion Shows a Nominal Enrichment in YA Group

**Figure 3:**
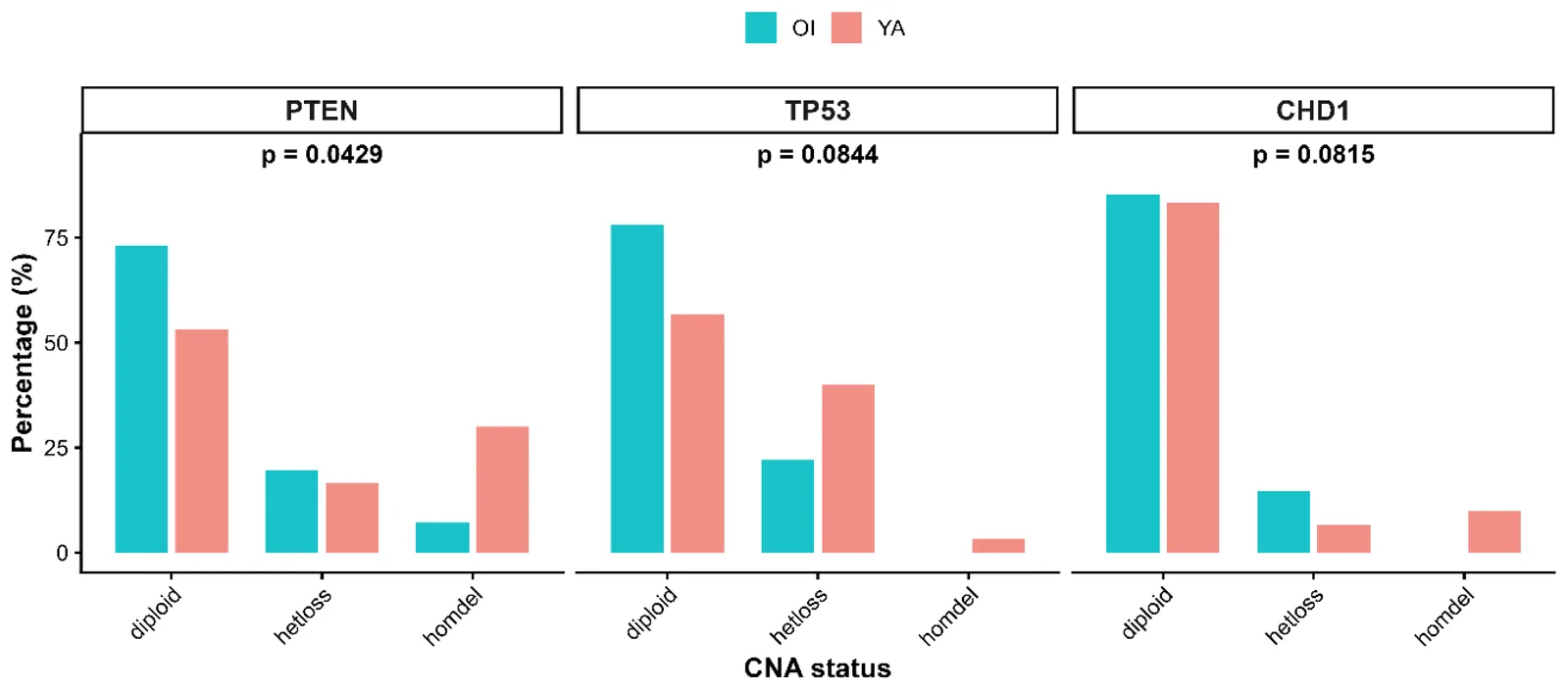
Copy number alteration profiles of key prostate cancer driver genes in YA and OI tumors. Bar plots show copy number alteration (CNA) states for *PTEN*, *TP53*, and *CHD1* in YA and OI tumors. CNA states were classified as diploid, heterozygous loss, or homozygous deletion. Group differences were evaluated using Fisher’s exact test. *p* values shown are nominal *p* values. YA: Young aggressive; OI: Old indolence-associated.

Given the strong association between chromosomal instability and aggressive disease, we next evaluated major PCa drivers for CNAs in YA and OI tumors. We focused on three key PCa driver genes: *PTEN*, *TP53*, and *CHD1*. *PTEN* copy number status showed a nominal difference between groups (Fisher’s exact test, nominal *p* = 0.0429) (Figure 3), but this association did not remain statistically significant after Benjamini–Hochberg correction (FDR-adjusted *p* = 0.0845). *TP53* and *CHD1* CNA distributions were also not significant after correction (nominal *p* = 0.0845 and 0.0815; FDR-adjusted *p* = 0.0845 for both). Homozygous *PTEN* deletion was observed in 30.0% of YA tumors (9/30) compared with 7.3% of OI tumors (3/41). In an exploratory binary comparison of homozygous *PTEN* deletion versus all other CNA states, the difference was nominally significant (Fisher’s exact test, *p* = 0.0220; odds ratio = 5.30). This secondary comparison was not included in the prespecified driver-gene multiple-comparison family and is therefore interpreted as exploratory. Thus, the *PTEN* CNA pattern represents a nominally enriched feature of the predefined YA phenotype rather than an FDR-significant difference.

### 2.5. AR Signaling Does Not Distinguish YA and OI Tumors

Because AR signaling is a central driver of PCa biology, we evaluated multiple AR-related features between YA and OI tumors, including AR score, AR protein abundance, AR mRNA expression, AR-V7 ratio, and AR-V7 positivity. No significant differences were observed across any AR-related metric (all *p* > 0.05), suggesting that canonical AR signaling does not substantially differ between YA and OI tumors. These AR-related findings should be interpreted in light of the available treatment metadata. Although neoadjuvant treatment was recorded as “No” for all 71 tumors, the available TCGA clinical field does not provide sufficient information to exclude previous androgen-deprivation therapy or other systemic treatment with certainty. Therefore, treatment-related effects on AR-associated measurements cannot be completely excluded.

### 2.6. YA and OI Tumors Exhibit Marked Transcriptomic Divergence

**Figure 4:**
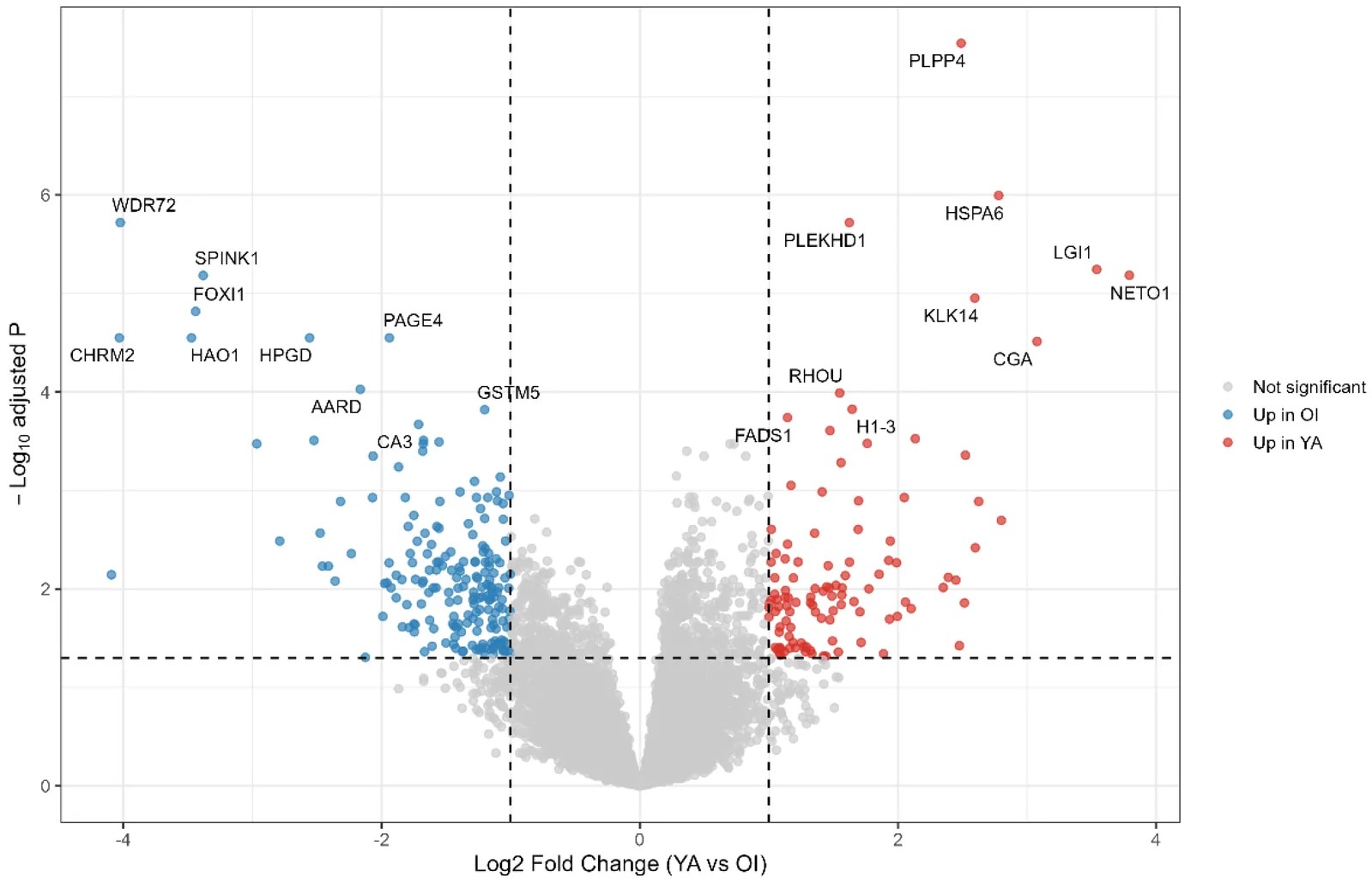
Transcriptomic differences between YA and OI prostate tumors. Volcano plot showing differential gene expression between YA and OI tumors. Red points indicate genes upregulated in YA, blue points indicate genes upregulated in OI, and gray points indicate non-significant genes. Dashed vertical lines indicate log_2_ fold-change thresholds (±1), and the horizontal dashed line indicates the FDR significance threshold (adjusted *p* = 0.05). Labeled genes correspond to the most statistically significant differentially expressed genes.

To characterize transcriptomic differences between the predefined YA and OI phenotypes, we performed differential expression analysis between YA and OI tumors using RNA-seq data from the 71-sample cohort. After protein-coding and low-expression filtering, 16,115 genes were retained for analysis. Differential expression analysis identified 1083 significantly dysregulated genes between YA and OI tumors at FDR-adjusted *p* < 0.05, indicating extensive transcriptomic divergence between the two phenotypic groups (Figure 4).

To prioritize genes with both statistical and biological relevance, we applied an additional effect-size threshold of absolute log_2_ fold-change ≥ 1. This yielded 300 stringently filtered differentially expressed genes (DEGs). Among the 300 differentially expressed genes, 110 showed higher expression in YA tumors, whereas 190 showed higher expression in OI tumors, corresponding to positive and negative log_2_ fold changes, respectively. This asymmetric distribution suggests that the YA phenotype may be characterized predominantly by widespread transcriptional repression rather than broad transcriptional activation. These 300 DEGs formed the basis for the downstream integrative RNA–CNA analysis, which subsequently identified and prioritized a 40-gene RNA–CNA concordant gene set for further analysis (Supplementary Table S3).

### 2.7. Integrative RNA–CNA Analysis Identifies a 40-Gene Exploratory RNA–CNA-Concordant Gene Set

**Figure 5:**
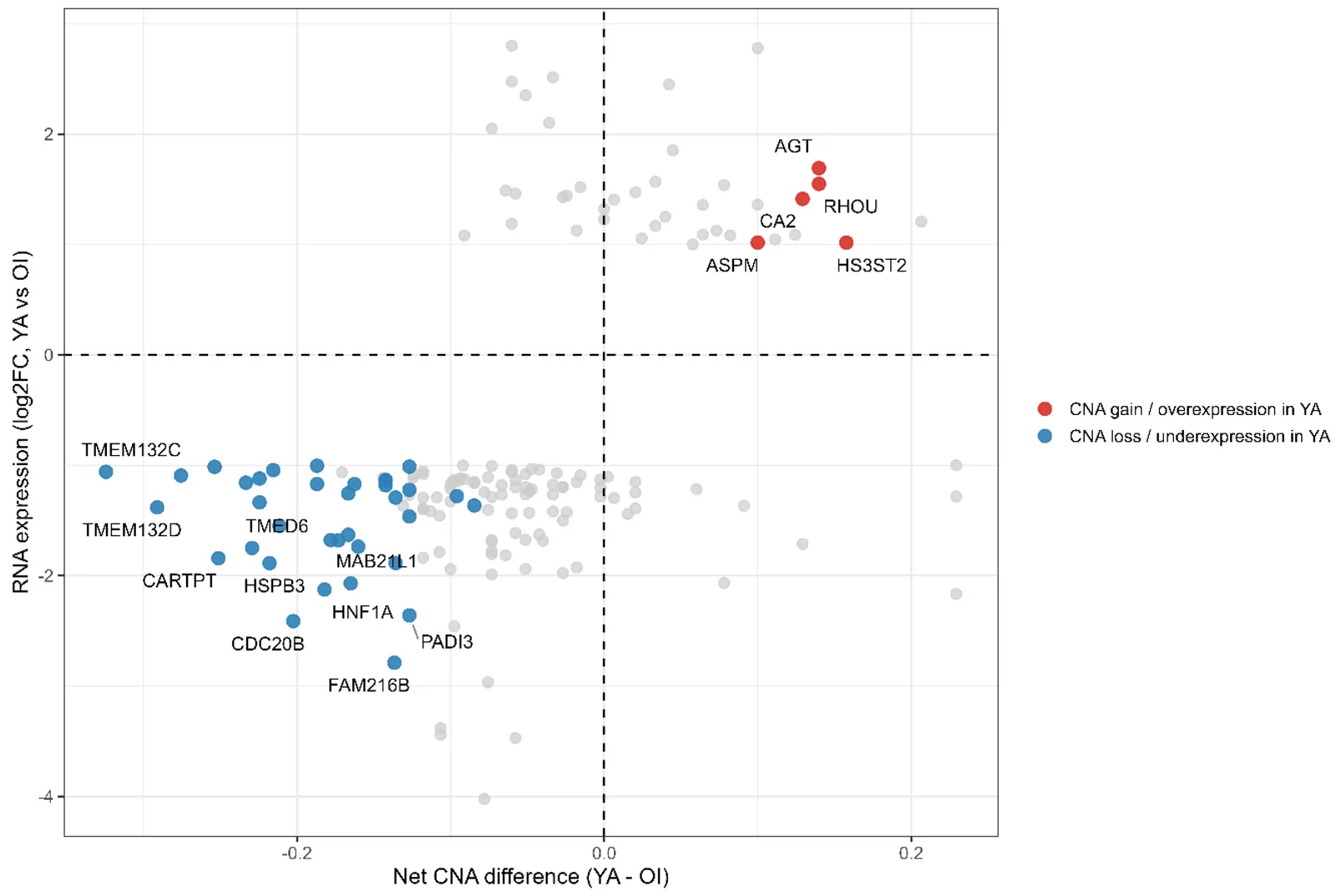
Integrative RNA–CNA analysis identifies prioritized exploratory concordant genes. Scatter plot showing the relationship between differential gene expression (log_2_ fold change, YA vs. OI) and net CNA difference (YA − OI) across RNA–CNA concordant genes. Gray points represent broad concordant genes not meeting strict filtering criteria. Colored points indicate prioritized exploratory RNA–CNA-concordant genes. Red points represent gain-associated overexpression in YA tumors, whereas blue points represent loss-associated underexpression in YA tumors. Dashed lines indicate zero RNA expression change (log_2_ fold change, YA vs. OI) and zero net CNA difference (YA − OI), defining the quadrants for concordant and discordant changes.

To determine whether transcriptomic differences between YA and OI tumors were associated with structural genomic alterations, we integrated RNA expression data with gene-level GISTIC2 copy number profiles. Among the 300 discovery-stage DEGs, 285 genes had available CNA data and were retained for integrative analysis. Gene-wise CNA comparisons between YA and OI tumors revealed no statistically significant differences after multiple-testing correction, likely reflecting limited power to detect individual gene-level CNA differences under strict FDR control. Despite this, exploratory analysis revealed multiple recurrent gain and loss events with clear directional consistency between CNA and gene expression changes. Broad RNA–CNA concordance analysis identified 168 of 285 genes (58.9%) with expression changes directionally consistent with CNA patterns (Figure 5). However, many of these genes exhibited only minimal differences in CNA frequencies between groups. To prioritize biologically meaningful associations, we applied a stricter effect-size threshold requiring at least a 10% difference in CNA frequency between YA and OI tumors. This reduced the number of concordant genes from 168 to 40, defining a subset of exploratory RNA–CNA-concordant genes. These 40 genes represented the prioritized exploratory subset used for subsequent functional, network, and clinical-association analyses (Table 1).

### 2.8. Sensitivity Analysis Adjusting for Tumor Purity

Because tumor purity differed significantly between YA and OI tumors and pathological stage showed clinically relevant between-group differences, we evaluated the robustness of the prioritized transcriptomic findings using three progressively adjusted DESeq2 models. The first model included tumor purity as a covariate, the second included tumor purity and pathological T stage (pT), and the third included tumor purity, pT, and nodal status (N). The sensitivity analyses were restricted to the 40 genes identified in the primary integrative RNA–CNA analysis and were performed to evaluate the robustness of the original prioritized gene set rather than to redefine it.

In the tumor-purity-adjusted model, 39 of the 40 genes (97.5%) retained the same direction of differential expression. Twelve genes (30.0%) remained statistically significant after adjustment (FDR < 0.05), and eight genes (20.0%) retained both the predefined statistical and biological criteria (FDR < 0.05 and |log_2_FC| ≥ 1): FAM216B, CARTPT, CA2, C15orf48, AGT, ELMOD1, RHOU, and HS3ST2 (Supplementary Table S4). When pT was additionally included as a covariate, RHOU was the only gene that retained both criteria, and the same gene retained both criteria in the model additionally adjusted for nodal status (N). Thus, although adjustment for tumor purity largely preserved the direction of the expression differences, progressive adjustment for clinicopathological covariates substantially reduced the number of genes meeting the predefined statistical and effect-size criteria. These findings support interpreting the 40-gene set as an exploratory RNA–CNA-concordant gene set, while individual genes that remain significant after covariate adjustment may represent more robust candidates for further validation. Complete results for all three sensitivity models are provided in Supplementary Table S5.

### 2.9. Exploratory Age-Stratified Analysis Within Gleason Strata

To further evaluate the potential contribution of age within comparable tumor-grade strata, we performed exploratory age-stratified analyses of the 40 prioritized genes. Within Gleason ≤ 7 tumors, 82 younger and 41 older tumors were compared, whereas within Gleason ≥ 8 tumors, 30 younger and 18 older tumors were compared. Complete results are provided in Supplementary Table S1. Within Gleason ≤ 7 tumors, 12 of the 40 genes showed nominal evidence of differential expression (*p* < 0.05), although only AGT remained significant after Benjamini–Hochberg correction (log_2_FC = −1.24, adjusted *p* = 0.028). Within Gleason ≥ 8 tumors, 10 genes showed nominal evidence of differential expression, with five remaining significant after correction: MCF2, SLITRK6, CRTAC1, SLC6A14, and AGT. These findings indicate that several expression differences observed in the primary YA-versus-OI comparison remain detectable within individual Gleason strata, while their magnitude and statistical support vary according to tumor grade.

PTEN CNA status was also evaluated within each Gleason stratum. No significant difference in the overall PTEN CNA distribution was observed within Gleason ≤ 7 tumors (Fisher’s exact test, *p* = 0.314) or Gleason ≥ 8 tumors (*p* = 0.092). Similarly, the comparison of homozygous PTEN deletion versus non-homozygous deletion was not significant within Gleason ≤ 7 tumors (*p* = 1.000) or Gleason ≥ 8 tumors (*p* = 0.0669). Thus, the PTEN difference observed in the primary comparison should be interpreted as a feature of the predefined extreme-phenotype comparison rather than as an age-independent association demonstrated within Gleason strata.

### 2.10. External Validation of the Expression Patterns of Prioritized Genes

External validation was performed using the independent RNA-sequencing cohort GSE229904 (Table 2). Of the 40 genes comprising the prioritized TCGA-derived RNA–CNA concordant gene set, 37 (92.5%) were available for evaluation, of which 27 (73.0%; 67.5% of the complete gene set) retained the same direction of differential expression observed in the TCGA discovery cohort. Eleven genes showed nominal evidence of differential expression (*p* < 0.05), including CARTPT, ELMOD1, F10, HSPB3, HSPB8, MAB21L1, MT1M, MYOCD, PLA2G2C, PLAC9, and RHOU. Ten of these preserved the original direction, whereas CARTPT showed an opposite pattern.

After Benjamini–Hochberg correction, none of the genes reached FDR-adjusted significance. However, 10 genes showed both directional concordance and nominal significance, and 8 additionally showed an absolute log_2_ fold change ≥ 1: ELMOD1, HSPB3, HSPB8, MAB21L1, MT1M, MYOCD, PLA2G2C, and PLAC9. Notably, although MT1M showed directional concordance and nominal significance in the external cohort, it did not retain the same expression direction after tumor-purity adjustment in the TCGA sensitivity analysis, further illustrating the limited overlap between external directional concordance and covariate-robust differential expression.

Overall, these findings provide exploratory evidence of partial expression-level consistency but not definitive validation of the complete gene set, particularly given the absence of FDR-significant genes and the limited number of YA tumors (n = 7). Because comparable gene-level copy-number data were unavailable, validation was restricted to gene expression and did not independently validate the CNA component or a causal RNA–CNA relationship. Notably, only ELMOD1 overlapped between the eight genes showing directional concordance with nominal significance and the eight genes retaining both predefined criteria after tumor-purity adjustment. This limited overlap further indicates that external directional concordance and covariate-robust differential expression represent distinct exploratory dimensions of evidence.

### 2.11. Copy-Number Losses Predominate Among RNA–CNA-Concordant Genes

**Figure 6:**
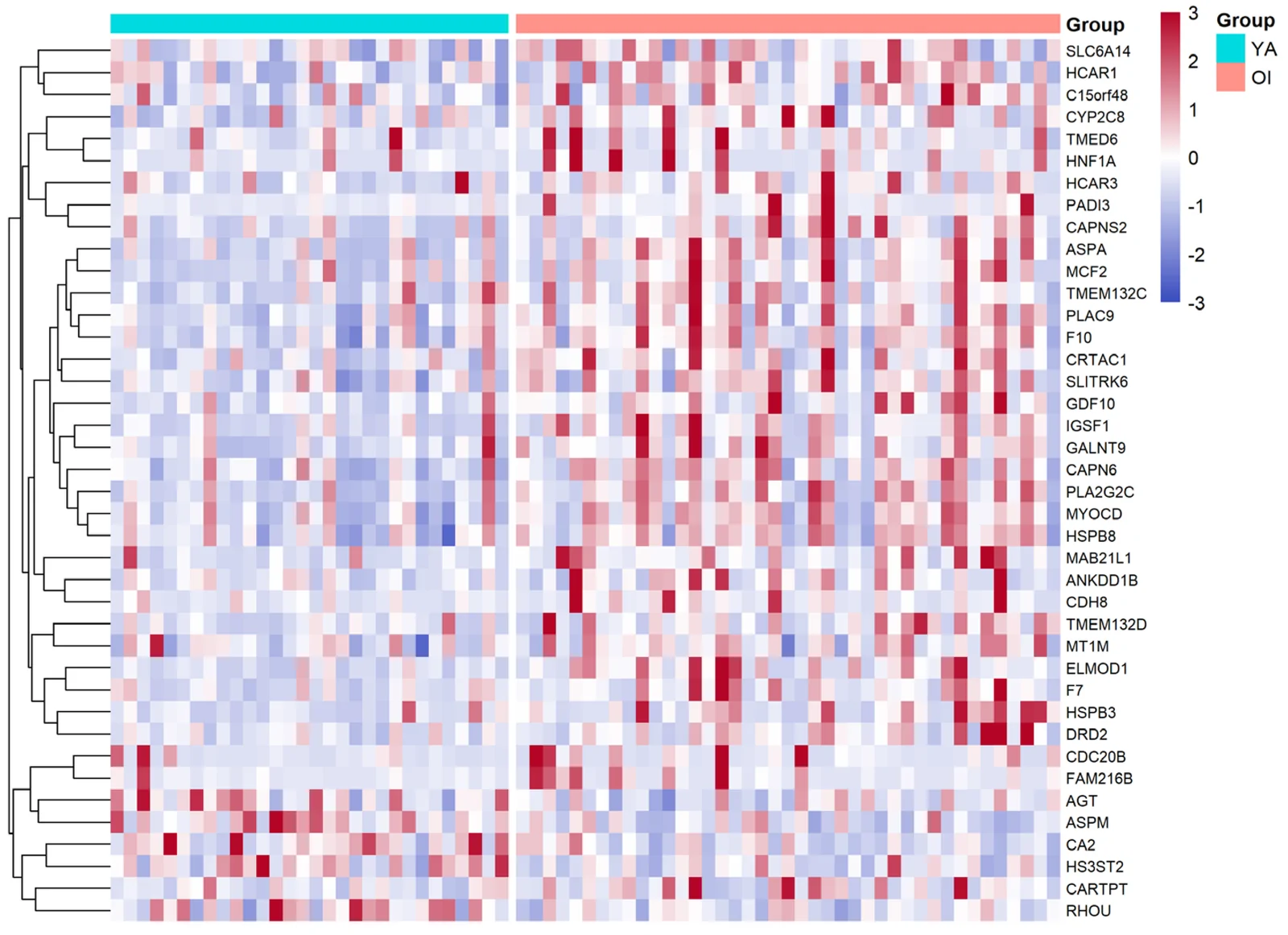
Expression landscape of strict RNA–CNA concordant genes in YA and OI tumors. Heatmap showing normalized expression profiles of the 40 prioritized exploratory RNA–CNA-concordant genes across YA and OI tumors. Gene expression values were log-transformed and standardized as row-wise Z-scores. Red indicates higher relative expression, white intermediate expression, and blue lower relative expression. YA: Young aggressive; OI: Old indolence-associated.

Analysis of the final 40-gene RNA–CNA gene set revealed a striking imbalance between gain and loss of transcriptional changes. Most strict concordant genes (35/40) showed reduced expression in YA tumors accompanied by increased copy-number loss frequency in YA tumors. In contrast, only 5 genes exhibited gain-associated overexpression (Figure 6). Among the most prominent loss-associated genes were *CDC20B*, *HSPB3*, *FAM216B*, *TMEM132C*, *TMEM132D*, *MYOCD*, and *HNF1A*, all of which displayed reduced expression in YA tumors together with increased copy-number loss frequency. Among gain-associated genes, *CA2*, *RHOU*, *ASPM*, and *AGT* showed increased gain frequency in YA tumors accompanied by elevated expression. These findings indicate that copy-number loss was the predominant CNA pattern among the exploratory RNA–CNA-concordant genes.

### 2.12. Exploratory Associations Between FGA and Expression of the Prioritized Genes

**Figure 7:**
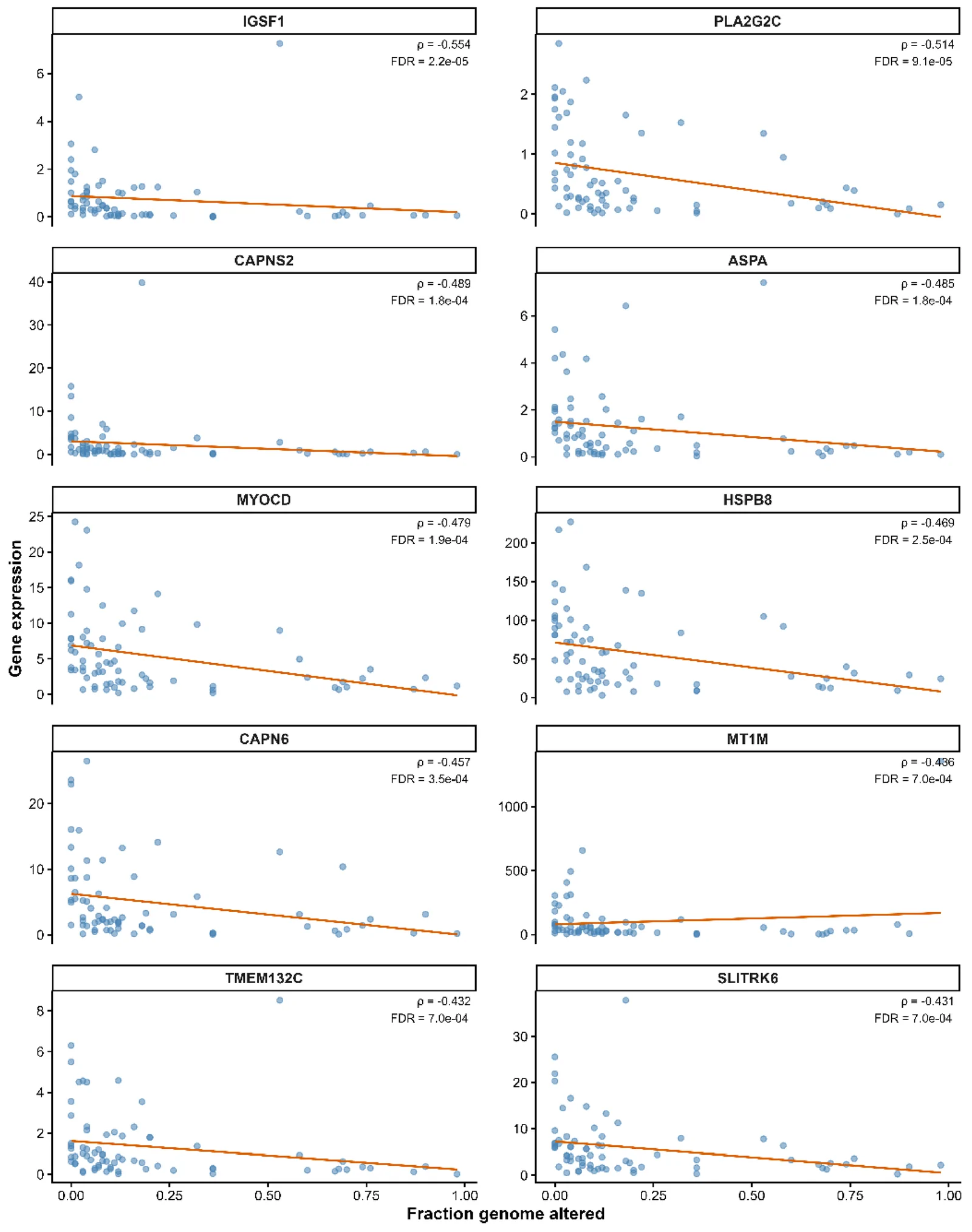
Exploratory associations between fraction genome altered (FGA) and expression of prioritized genes. Scatterplots show the relationship between FGA and normalized expression for the top 10 genes identified in the unadjusted analysis. Each point represents an individual tumor sample. Genes were ranked according to the absolute Spearman correlation coefficient (ρ). Spearman correlation coefficients and FDR-adjusted *p* values from the unadjusted analysis are shown in each panel. Linear trend lines are shown for visualization only. Tumor-purity-adjusted analyses of all 40 genes are provided in Supplementary Table S6.

To further investigate the clinical and genomic associations of the 40-gene exploratory RNA–CNA-concordant gene set, we evaluated correlations between gene expression and preoperative PSA or fraction genome altered (FGA). The FGA analysis was considered exploratory because the 40 genes had been selected from the same TCGA cohort using expression and CNA patterns, making these subsequent correlations a post-selection analysis. In the unadjusted analysis, 25 of the 40 genes showed FDR-adjusted associations with FGA (Figure 7). However, because tumor purity differed between the YA and OI groups, we additionally evaluated the FGA–expression relationships in the 62 tumors with available tumor-purity measurements using linear regression models adjusted for tumor purity. None of the 40 genes retained FDR significance after tumor-purity adjustment (Supplementary Table S6). Thus, the observed FGA–expression relationships should be interpreted as exploratory associations that may be partly influenced by tumor purity rather than as independent associations with genomic instability.

In the unadjusted analysis, most of the FDR-significant correlations were negative, with IGSF1 showing the strongest association (Spearman’s ρ = −0.536, FDR = 5.69 × 10^−5^), followed by PLA2G2C (ρ = −0.501), CAPNS2 (ρ = −0.499), HSPB8 (ρ = −0.478), and ASPA (ρ = −0.477). However, none of these associations remained FDR-significant after adjustment for tumor purity, indicating that the unadjusted relationships may be sensitive to differences in tumor composition. These findings therefore provide exploratory evidence of an association between FGA and expression within the prioritized gene set, but do not establish tumor-purity-independent relationships.

### 2.13. Functional Characterization of the RNA–CNA-Associated Gene Set

To investigate the biological functions represented by the final 40-gene RNA–CNA concordant gene set, functional enrichment analyses were performed. This 40-gene set comprised 35 genes showing concordant copy-number loss-associated downregulation and five genes showing concordant copy-number gain-associated upregulation in YA tumors. Accordingly, enrichment analyses were performed using the complete 40-gene exploratory RNA–CNA-concordant gene set.

**Figure 8:**
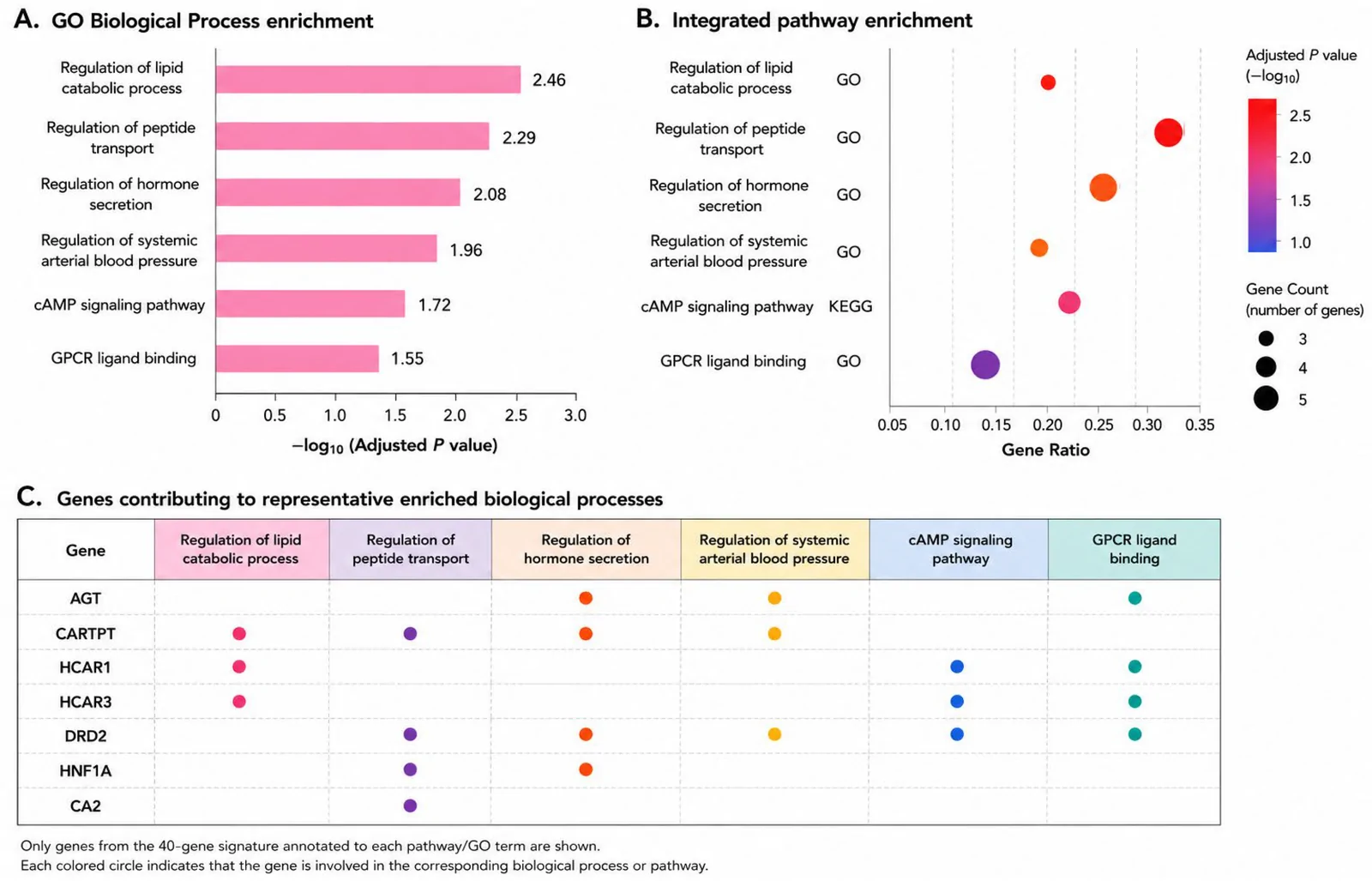
Functional enrichment landscape of the RNA–CNA-associated gene set. Functional enrichment analysis of the 40-gene exploratory RNA–CNA-concordant gene set in YA tumors. (**A**) GO Biological Process enrichment. (**B**) Representative pathway enrichment across multiple annotation databases. (**C**) Gene–pathway involvement matrix showing the genes contributing to representative enriched biological processes and pathways. GO, Gene Ontology; KEGG, Kyoto Encyclopedia of Genes and Genomes.

Gene Ontology (GO) Biological Process enrichment analysis identified biological processes primarily related to metabolic regulation, endocrine homeostasis, and physiological regulation (Figure 8). The most significantly enriched terms included regulation of lipid catabolic process, regulation of peptide transport, regulation of synapse organization, adipogenesis, response to peptide hormone, regulation of hormone secretion, regulation of systemic arterial blood pressure, and response to toxic substances.

Complementary pathway enrichment analyses using KEGG, Reactome, Hallmark, and WikiPathways consistently reproduced the biological themes identified by GO analysis. KEGG highlighted pathways related to linoleic acid metabolism, arachidonic acid metabolism, cAMP signaling, pancreatic secretion, and nitrogen metabolism, whereas Reactome identified pathways involved in peptide processing, protein transport, fibrin clot formation, and protein γ-carboxylation. Hallmark analysis additionally supported enrichment of estrogen response, complement activation, fatty acid metabolism, xenobiotic metabolism, coagulation, and IL2/STAT5 signaling. These results indicate that the 40-gene exploratory RNA–CNA-concordant gene set is predominantly associated with metabolic, homeostatic, and hormone-responsive biological processes rather than canonical proliferative pathways.

### 2.14. Protein–Protein Interaction and Network Topology Analysis

**Figure 9:**
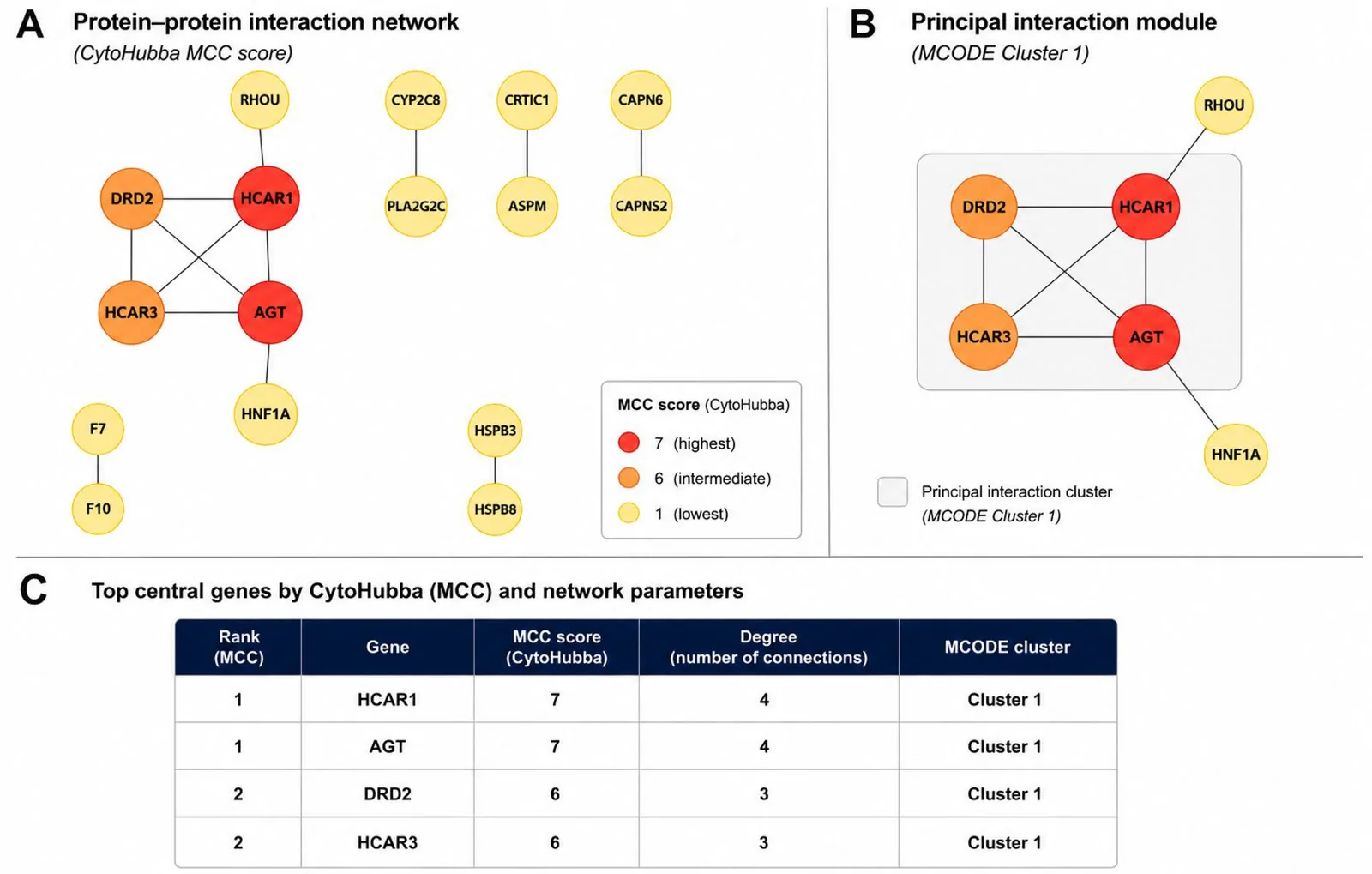
Identification of central genes within the RNA-associated copy number alteration (CNA) gene set through a protein–protein interaction network. (**A**) Protein–protein interaction network. (**B**) Principal interaction module identified by Molecular Complex Detection (MCODE). (**C**) Ranking of the highest-scoring hub genes according to the CytoHubba Maximal Clique Centrality (MCC) algorithm.

Following functional enrichment analysis, the protein–protein interaction (PPI) network of the RNA–CNA-associated gene set was explored to identify its central network components (Figure 9). The resulting interaction network consisted of several interconnected subnetworks rather than a single highly connected interactome. Functional module detection using the MCODE algorithm identified one principal interaction module containing *HCAR1*, *HCAR3*, *DRD2*, and *AGT*.

Network visualization and topology analysis were performed in Cytoscape, whereas node centrality was evaluated using the Maximal Clique Centrality (MCC) algorithm implemented in the CytoHubba plugin. CytoHubba identified *HCAR1* and *AGT* as the highest-ranked hub genes (MCC = 7), followed by *DRD2* and *HCAR3* (MCC = 6). These four genes also exhibited the highest degree values within the interaction network and comprised the principal MCODE cluster. The concordance between network topology, hub-gene ranking, and module detection supports *HCAR1*, *AGT*, *DRD2*, and *HCAR3* as the central network nodes of the transcriptionally repressed molecular gene set associated with YA prostate tumors.

## 3. Discussion

PCa is characterized by remarkable biological heterogeneity, ranging from indolent tumors with prolonged clinical stability to highly aggressive cancers associated with rapid progression and poor clinical outcomes. Although age is an important epidemiological factor in PCa, molecular heterogeneity may also be observed across clinically defined patient groups. In this context, comparing phenotypically contrasting tumors may help identify molecular features associated with tumor aggressiveness and disease biology [7,8,10]. By comparing two clinically divergent phenotypes, YA and OI tumors, our integrative analyses identified differences in genomic architecture, transcriptomic organization, and molecular subtype distribution. Collectively, these findings suggest molecular differences between the YA and OI phenotypes, including differences in genomic alteration burden and RNA–CNA concordance.

Previous studies have reported molecular and clinical heterogeneity across age groups in PCa, including differences in genomic alterations, transcriptional profiles, and clinical presentation [7,8]. However, the extent to which these differences reflect age itself, tumor aggressiveness, or the interaction between these factors remains incompletely understood [2,3,10]. Our findings contribute to this broader understanding by identifying molecular differences between two predefined extreme clinical phenotypes, YA and OI, rather than by establishing an independent age-related molecular effect.

Compared with OI tumors, YA tumors exhibited higher genomic instability, a higher nominal frequency of homozygous PTEN deletion, distinct miRNA cluster distribution, and widespread transcriptomic alterations, whereas androgen receptor (AR) signaling remained largely preserved. The convergence of these molecular features should therefore be interpreted as differences between the predefined YA and OI phenotypes rather than as age-specific effects independent of tumor grade or differences attributable to enhanced AR signaling. Instead, our findings raise the possibility that differences in structural genomic alteration burden may be associated with the transcriptional differences observed between the YA and OI phenotypes.

Characterizing the genomic features associated with these phenotypic differences may help clarify the molecular features associated with the YA phenotype. In the present study, YA tumors exhibited a higher FGA, together with a higher nominal frequency of homozygous *PTEN* deletion. The higher FGA, together with the higher nominal frequency of homozygous *PTEN* deletion, is consistent with a greater burden of structural genomic alterations in the YA group. As a quantitative measure of chromosomal gains and losses across the genome, FGA has consistently been associated with aggressive clinicopathological characteristics and adverse clinical outcomes in PCa, highlighting its value as a surrogate marker of chromosomal instability [6,11].

This observation is further supported by the higher nominal frequency of homozygous *PTEN* deletion in YA tumors, although the overall *PTEN* CNA distribution did not remain significant after Benjamini–Hochberg correction. *PTEN* is one of the most frequently inactivated tumor suppressor genes in PCa and a central regulator of the *PI3K*/*AKT* signaling pathway [12]. Beyond its canonical role in cell proliferation and survival, *PTEN* contributes to the maintenance of genomic integrity, and *PTEN* loss has been associated with chromosomal instability and aggressive disease in PCa [12,13].

Our integrative RNA–CNA analysis identified a predominance of copy-number loss-associated transcriptional repression among the exploratory concordant genes. Remarkably, 87.5% (35/40) of the prioritized RNA–CNA-concordant genes exhibited transcriptional downregulation together with increased copy-number loss frequency in YA tumors. Since CNAs can influence gene dosage and gene-expression variability, such concordant patterns may reflect relationships between structural genomic alterations and transcriptional states [14,15]. However, because no individual gene-level CNA differences between YA and OI tumors remained statistically significant after multiple-testing correction in our cohort, the observed RNA–CNA concordance should be interpreted as exploratory and hypothesis-generating rather than as evidence of a CNA-mediated mechanism.

The molecular differences between the YA and OI phenotypes may also be considered in the broader context of biological aging. The OI phenotype occurs in an older patient population in which, under the cumulative influence of age-associated biological processes, including chronic low-grade inflammation (inflammaging), cellular senescence, stromal remodeling, and progressive loss of tissue homeostasis may contribute to the observed molecular context [16,17]. The predominance of copy-number loss-associated repression observed in our integrative analyses is consistent with an association between genomic alteration burden and widespread transcriptional differences. Copy-number losses were associated with coordinated repression of genes involved in metabolism, hormone responsiveness, peptide transport, and cellular homeostasis. This interpretation is consistent with previous evidence linking chromosomal instability to tumor evolution through increased genomic heterogeneity, clonal selection, and changes in regulatory networks that sustain malignant progression [18,19]. Although these interpretations require experimental validation, our integrative analyses identify biological programs that warrant further investigation in experimental models to determine their potential relevance of the YA phenotype.

An additional finding supporting the molecular divergence between YA and OI tumors was the significant difference observed in TCGA-derived miRNA cluster distribution, whereas mRNA, methylation, and SCNA molecular classifications remained comparable. miRNAs are major post-transcriptional regulators capable of simultaneously modulating multiple signaling pathways associated with carcinogenesis [20,21]. Consequently, alterations affecting global miRNA regulatory programs may produce broad phenotypic effects that extend beyond changes in individual gene expression [22]. The differential distribution of miRNA molecular subtypes observed in YA tumors therefore suggests that differences in post-transcriptional regulatory programs may be associated with the transcriptomic differences observed in the YA phenotype.

These findings are consistent with our previous work demonstrating that age is associated with coordinated alterations in promoter methylation and expression of multiple miRNAs in PCa, supporting the existence of age-dependent epigenetic remodeling of miRNA regulatory programs [21]. When considered together with the present results, these observations suggest that differences in miRNA regulatory programs may extend beyond chronological age and may also be associated with the predefined YA phenotype. Future studies integrating miRNA expression, epigenetic regulation, and target-gene networks will be necessary to clarify their potential relevance to the YA phenotype.

Despite the marked genomic and transcriptomic differences observed between YA and OI tumors, *AR* signaling metrics did not significantly differ between both groups. Thus, the observed molecular differences between the YA and OI phenotypes do not appear to be explained by differences in canonical *AR* signaling alone, while other molecular features, including genomic alteration burden and copy-number-associated transcriptional patterns, may also be relevant to the observed molecular divergence. This observation is consistent with large-scale molecular profiling studies indicating that aggressive PCa involves multiple genomic and epigenomic alterations beyond canonical AR signaling [23]. In agreement with this concept, we previously indicate that *AR* expression is not substantially altered in PCa tissues from Mexican patients, although it correlates with androgen receptor CAG repeat length, supporting the notion that *AR* activity may be modulated by mechanisms beyond transcript abundance [24].

If canonical *AR* signaling does not account for the molecular divergence between YA and OI tumors, identifying the biological processes affected by the RNA–CNA concordant transcriptional changes become particularly relevant. Functional enrichment analyses therefore provide an opportunity to characterize the biological processes and cellular functions represented by the coordinated gene repression associated with aggressive disease. Nevertheless, pathway-level analyses provide only a global view of molecular alterations and may overlook biologically relevant genes that contribute through more specialized or context-dependent mechanisms. Consistent with this interpretation, functional enrichment analysis identified biological processes enriched among the RNA–CNA-associated gene set, providing a systems-level overview of the functional categories represented among the molecular differences observed between YA and OI prostate tumors. However, pathway-based analyses are designed to identify shared biological functions and therefore do not necessarily capture the biological relevance of every individual gene within a molecular gene set. Consequently, genes that do not contribute significantly to pathway enrichment may still participate in specialized or context-dependent regulatory mechanisms. This distinction is particularly relevant when comparing biologically divergent clinical phenotypes such as YA and OI tumors, where individual genes may participate in phenotype-associated molecular adaptations despite not defining the predominant biological pathways.

Among these genes, *HSPB8* represents the strongest biologically supported candidate. Using integrated transcriptomic analyses together with functional validation, Fu et al. (2025) [25] demonstrated that *HSPB8* acts as a tumor suppressor in PCa, showing reduced expression in tumor tissues and promoting cell proliferation, migration, and invasion following gene silencing through activation of the PI3K/AKT signaling pathway. Likewise, Feng et al. (2025) [26] highlighted the central role of small heat shock proteins in regulating proteostasis, stress adaptation, therapeutic resistance, and tumor progression in PCa. Since impairment of proteostasis is a recognized hallmark of biological aging, the downregulation of *HSPB8* observed in YA tumors raises the possibility that proteostasis-related processes may differ between the YA and OI phenotypes, warranting further investigation in experimental models.

Among the less-characterized candidates emerging from the integrative analysis, *RHOU* is of particular interest given its potential role in regulating cytoskeletal organization and cell signaling. *RHOU* encodes a Rho family GTPase that has been implicated in the regulation of actin cytoskeletal dynamics, cell morphology, and migratory behavior [27,28]. These functions are relevant to tumor biology, although the specific contribution of *RHOU* to the molecular phenotype observed in younger patients with PCa remains unclear. Its persistence across the sensitivity analyses and partial directional concordance in the independent cohort further support its consideration as a candidate for future investigation, while the lack of FDR-adjusted significance in the external cohort precludes definitive conclusions regarding its biological or clinical relevance.

Other genes within the 40-gene exploratory RNA–CNA-concordant gene set, including *CAPN6*, *CYP2C8*, *PLA2G2C*, and *CARTPT*, remain poorly characterized in the context of PCa. *CAPN6* has been associated with cytoskeletal organization, cell migration, and tumor progression in several malignancies, whereas *CYP2C8* belongs to the cytochrome P450 family involved in xenobiotic metabolism and has been reported to be expressed in prostate tumors, where it may influence drug metabolism and therapeutic response [29,30]. In contrast, little or no functional evidence is currently available linking *PLA2G2C* or *CARTPT* to prostate cancer biology. Rather than diminishing their potential biological relevance, the coordinated transcriptional repression of these genes in YA tumors, together with their integration within the RNA–CNA concordant gene set, supports their consideration as candidates for future functional studies. Their consistent co-repression suggests that they may be associated with biological programs represented in the YA phenotype, although experimental validation will be required to determine their potential functional relevance.

Beyond individual genes, the integrative analyses performed in the present study revealed that the molecular differences between YA and OI prostate tumors are organized into coordinated biological pathways and interaction networks. Functional enrichment analysis demonstrated that the RNA–CNA-associated gene set was primarily associated with lipid metabolism, peptide transport, hormone responsiveness, and cellular homeostasis. Rather than representing canonical proliferative or cell-cycle pathways, these findings suggest that the YA phenotype is associated with transcriptional differences involving metabolic and homeostatic processes [23,31].

Integration of functional enrichment, protein–protein interaction, and network topology analyses identified a set of interconnected genes associated with metabolic regulation, GPCR-mediated signaling, hormone responsiveness, and cellular homeostasis. Rather than representing isolated transcriptional events, the RNA–CNA-associated genes identified in YA tumors were enriched in interconnected biological pathways that the RNA–CNA-associated genes identified in YA tumors are enriched in interconnected biological pathways that have been implicated in tumor metabolic regulation and prostate cancer progression [32]. Within the network constructed from the original 40-gene exploratory set, *HCAR1*, *AGT*, *DRD2*, and *HCAR3* occupied central topological positions. However, these network-level observations should be interpreted in light of the sensitivity analyses, in which several of these genes did not retain the predefined differential-expression criteria after adjustment for tumor purity and pathological covariates. *HCAR1* is a lactate receptor involved in metabolic adaptation and lactate-mediated signaling, processes that have been associated with cancer progression and immune modulation [33,34]. *AGT* participates in the renin–angiotensin system, which has been associated with prostate cancer progression through its effects on proliferation, angiogenesis, inflammation, and tumor microenvironment remodeling [35]. *DRD2* belongs to the dopamine receptor family of G protein-coupled receptors involved in neuroendocrine signaling pathways that contribute to prostate cancer biology [36]. Although the role of *HCAR3* in prostate cancer remains poorly characterized, its recurrent identification across independent analytical approaches suggests that it may warrant further investigation within the transcriptionally repressed interaction network associated with the YA phenotype. The network-level organization of these genes further supports the interpretation that the molecular differences observed in the YA phenotype involve coordinated changes across metabolic and signaling-related genes rather than isolated gene-level alterations.

Interestingly, the biological functions represented by these hub genes are highly coherent with the enriched pathways identified in our study. *HCAR1* and *HCAR3* encode hydroxycarboxylic acid G protein-coupled receptors involved in metabolic sensing and cellular adaptation, with *HCAR1* functioning as the canonical lactate receptor [33,34]. *AGT*, the precursor of angiotensin peptides, participates in hormonal regulation, vascular homeostasis, and tissue remodeling, whereas *DRD2* mediates dopaminergic signaling through GPCR-dependent pathways that have increasingly been implicated in cancer cell proliferation, metabolic adaptation, and therapeutic response [35,37,38]. The convergence of these functional annotations and network relationships highlights metabolic and homeostatic processes as areas for further investigation in the YA phenotype. From a translational perspective, current prognostic models are largely based on clinicopathological variables, including PSA levels, Gleason grade, and tumor stage; however, these parameters do not fully capture the extensive molecular heterogeneity underlying PCa progression and clinical outcome [39]. The combined analysis of transcriptomic and copy-number alterations provides an exploratory framework for characterizing the molecular features of the YA phenotype.

The exploratory nature of the prioritized gene set is further underscored by the sensitivity analyses. Although most genes retained their direction of differential expression after tumor-purity adjustment, only 8 of the 40 genes retained both the predefined FDR and effect-size criteria, and only one gene retained both criteria after additional adjustment for pT and N. Moreover, the genes showing the strongest external directional concordance showed limited overlap with those retaining the original criteria after purity adjustment. Therefore, the 40-gene set and the HCAR1–AGT–DRD2–HCAR3 network module should not currently be considered a validated biomarker panel. Instead, they represent exploratory candidate features that warrant independent validation and, ultimately, prospective assessment before any biomarker application can be considered [40,41]. Importantly, the attenuation of several transcriptional differences after adjustment for tumor purity and pathological stage suggests that part of the observed YA–OI expression differences may reflect differences in tumor composition or disease stage. Conversely, the persistence of selected differences across progressively adjusted models identifies a smaller subset of candidates that may warrant further investigation as potentially more robust features of the YA phenotype.

As with any integrative retrospective study, the present findings should be interpreted within the context of the study design. Because the YA and OI groups were defined jointly by age and tumor grade, the present design does not allow the independent contribution of age to be disentangled from tumor grade. Similarly, the OI designation reflects a predefined combination of older age and lower tumor grade and should not be interpreted as clinically validated indolence, as recurrence, metastatic progression, and longitudinal clinical outcomes were not incorporated into the phenotype definition. Therefore, the molecular differences identified in this study should be interpreted as differences between two predefined extreme clinical phenotypes rather than age-specific effects independent of tumor aggressiveness. An additional limitation concerns treatment history. In the available TCGA clinical metadata, neoadjuvant treatment was recorded as “No” for all 71 tumors in the primary cohort, with complete metadata for this variable. However, these clinical data do not comprehensively capture all forms of prior systemic therapy and cannot exclude previous androgen-deprivation therapy with certainty. This limitation is particularly relevant to the interpretation of *AR*-related measurements and bulk transcriptomic profiles. The analyses were performed using a retrospective TCGA-PRAD cohort, and the molecular associations identified in this study require validation in independent patient populations and functional experimental models. Likewise, the network-level interactions identified through integrative computational analyses should be interpreted as biologically supported hypotheses rather than direct evidence of causality. Nevertheless, by integrating transcriptomic, copy-number, clinical, functional enrichment, and network analyses within an extreme-phenotype design, our study provides a systems-level characterization of the molecular features distinguishing YA from OI prostate tumors. Taken together, the integrative analyses provide an exploratory framework for future mechanistic studies and independent validation of the molecular candidates and biological pathways identified in the YA phenotype.

## 4. Materials and Methods

### 4.1. Study Design and Cohort Selection

This study was designed as a retrospective in silico multi-omics analysis to investigate molecular differences between two predefined extreme clinical phenotypes: YA and OI prostate tumors. These phenotypes were defined jointly according to age and pathological Gleason score. Importantly, because age and pathological Gleason score were jointly used to define the study groups, this design does not allow the independent effects of age and tumor aggressiveness to be disentangled. Accordingly, all downstream analyses were interpreted as comparisons between two predefined extreme clinical phenotypes rather than estimates of age-specific molecular effects independent of tumor grade. Clinical, transcriptomic, and genomic data were obtained from The Cancer Genome Atlas Prostate Adenocarcinoma (TCGA-PRAD) cohort, data accessed on 2 July 2026. Tumor tissues were collected, processed, and stored according to TCGA standard operating procedures, which include centralized pathology review, snap-freezing of tumor samples, and storage in biorepositories under controlled conditions. RNA sequencing and copy number alteration data were generated from these biospecimens following TCGA quality assurance protocols. RNA sequencing data from TCGA-PRAD initially included 554 samples. To ensure cohort homogeneity and avoid confounding effects from non-primary lesions, the dataset was restricted to primary tumor samples only, yielding 501 tumors. Clinical metadata were then evaluated to identify cases with complete information for age at diagnosis and pathological Gleason score, resulting in 283 tumors eligible for phenotype stratification (Supplementary Table S7). The age thresholds of 58 and 67 years were selected after examining the distribution of age and pathological Gleason score among the 283 tumors with complete clinical information. Rather than representing prespecified clinical cutoffs, these thresholds were pragmatically selected to maximize age and phenotypic separation while retaining adequate sample sizes for downstream analyses. YA tumors were defined as patients aged ≤ 58 years with pathological Gleason score ≥ 8, whereas OI tumors were defined as patients aged ≥ 67 years with pathological Gleason score ≤ 7. The term “indolence-associated” refers specifically to this predefined combination of older age and lower pathological Gleason score and does not imply clinically validated indolence based on recurrence, metastasis, or longitudinal outcomes. This strategy yielded 71 tumors, including 30 YA tumors (11 Gleason 8 and 19 Gleason 9) and 41 OI tumors (8 Gleason 6 and 33 Gleason 7). Thus, these cohort-specific thresholds were used to define two extreme phenotypes and should not be interpreted as universal clinical or biological boundaries. Treatment history was assessed using the available TCGA clinical metadata. Neoadjuvant treatment was recorded as “No” for all 71 tumors included in the primary cohort, corresponding to 100% completeness for this variable. Because this metadata field does not comprehensively capture all forms of prior systemic therapy, absence of recorded neoadjuvant treatment was not interpreted as definitive evidence that all patients were completely treatment-naïve.

This study was assessed according to the Strengthening Reporting of Observational Studies in Epidemiology—Molecular Epidemiology (STROBE-ME) guidelines. The completed STROBE-ME checklist, indicating the location of each relevant item in the manuscript, is provided as Supplementary Table S8.

### 4.2. Clinical, Molecular, and Genomic Comparisons

Clinical and molecular features were compared between YA and OI groups to identify differences associated with the YA phenotype. Continuous variables analyzed included preoperative prostate-specific antigen (PSA), fraction genome altered (FGA), mutation burden, absolute ploidy, *AR* score, AR protein abundance, *AR* mRNA expression, and AR-V7 ratio. Categorical variables included SCNA cluster, mRNA cluster, methylation cluster, miRNA cluster, *PTEN* copy number status, *TP53* copy number status, *CHD1* copy number status, and AR-V7 presence. Continuous variables were compared using the Wilcoxon rank-sum test. Categorical variables were analyzed using Fisher’s exact test. For families of related comparisons, nominal *p* values were adjusted using the Benjamini–Hochberg procedure to control the false discovery rate (FDR), with statistical significance defined as an adjusted *p* value < 0.05. The clinical–pathological comparison family comprised five prespecified variables: preoperative PSA, pT stage (T3/T4), N status (N1), residual tumor status (R1/R2), and prior treatment. Age and Gleason score were not subjected to inferential testing because they were used to define the YA and OI study groups, respectively.

### 4.3. RNA Sequencing Processing and Differential Expression Analysis

RNA sequencing count data from TCGA-PRAD were used to characterize transcriptomic differences between YA and OI tumors. The raw RNA-seq dataset initially contained 60,660 genes across 554 samples. Gene annotation filtering was performed to retain protein-coding genes only, yielding 19,962 genes. To reduce technical noise and improve statistical power, low-expression genes were filtered using an expression threshold requiring a minimum count of 10 in at least 20% of samples. After filtering, 16,115 genes were retained for differential expression analysis. Differential expression analysis was performed using DESeq2 under a negative binomial generalized linear model with group status as the primary covariate: design = ~group. Comparisons were performed between YA and OI tumors, with positive log_2_ fold-change values indicating higher expression in YA tumors and negative values indicating higher expression in OI tumors. Statistical significance was defined using the Benjamini–Hochberg false discovery rate (FDR) correction with an adjusted *p*-value threshold of <0.05. To identify stringently filtered differentially expressed genes (DEGs) with both statistical and biological significance, an additional absolute log_2_ fold-change threshold of ≥1 was applied. Genes meeting both criteria (*padj* < 0.05 and |log_2_FC| ≥ 1) were retained for downstream integrative analyses. Because tumor purity differed between the YA and OI groups and pathological stage showed clinically relevant differences between groups, sensitivity analyses were performed to assess the robustness of the differential expression findings to potential confounding. Using the same count matrix, filtering criteria, normalization procedure, and DESeq2 framework as in the primary analysis, three progressively adjusted models were evaluated: (i) tumor purity as a covariate; (ii) tumor purity plus pathological T stage (pT); and (iii) tumor purity plus pT and nodal status (N). The analyses were restricted to the 40 genes prioritized in the primary integrative RNA–CNA analysis and were intended as sensitivity analyses rather than as re-selection procedures. For each model, log_2_ fold-change estimates and Benjamini–Hochberg-adjusted *p* values were compared with the predefined criteria used for the primary analysis. A gene was considered to retain the original DEG criteria when FDR-adjusted *p* < 0.05 and log_2_ fold-change ≥ 1. Complete results for the three sensitivity models are provided in Supplementary Table S5.

### 4.4. Exploratory Age-Stratified Analysis Within Gleason Strata

To evaluate whether transcriptomic differences remained detectable when age comparisons were performed within comparable tumor-grade strata, exploratory age-stratified analyses were conducted separately within Gleason ≤ 7 and Gleason ≥ 8 tumors. Within Gleason ≤ 7, 82 younger and 41 older tumors were available, whereas within Gleason ≥ 8, 30 younger and 18 older tumors were available. Differential expression of the 40 prioritized genes was evaluated using DESeq2 with age group as the explanatory variable within each Gleason stratum. Log_2_ fold changes, standard errors, Wald statistics, nominal *p* values, and Benjamini–Hochberg-adjusted *p* values were obtained for each gene. An additional interaction model including age group, Gleason stratum, and their interaction was used to identify genes for which the age-associated expression difference differed between Gleason strata. PTEN CNA status was evaluated separately within each Gleason stratum using Fisher’s exact test, considering both the three-level CNA classification and a binary comparison of homozygous deletion versus non-homozygous deletion. These analyses were exploratory sensitivity analyses and were not used to redefine the original 40-gene set or the primary YA and OI phenotypes. Complete results are provided in Supplementary Table S1. FGA was not subjected to the age-stratified within-Gleason analysis because the number of evaluable FGA measurements within the individual age-by-Gleason strata was insufficient for a reliable inferential comparison. The numbers of evaluable FGA measurements are reported in the corresponding methodological description.

### 4.5. Copy Number Alteration Analysis

Gene-level CNA data for TCGA-PRAD were obtained from UCSC Xena as GISTIC2 thresholded gene-level profiles. CNA states were encoded as follows:

−2 = deep deletion;

−1 = shallow deletion;

0 = diploid;

+1 = low-level gain;

+2 = amplification.

CNA analysis was restricted to the same 71 tumor samples used for transcriptomic comparisons (30 YA and 41 OI tumors). A total of 300 DEGs identified from RNA-seq analysis were queried in the CNA dataset. Of these, 285 genes were available in the GISTIC2 matrix and retained for integrative analysis. For each gene, CNA frequencies were calculated separately in YA and OI tumors. Gain and loss frequencies were defined as Gain = CNA > 0 and Loss = CNA < 0. Differences in CNA frequencies between groups were calculated as ΔGain = GainYA − GainOI and ΔLoss = LossYA − LossOI. Gene-level CNA frequencies between groups were compared using Fisher’s exact tests. *p*-values were adjusted for multiple testing using the Benjamini–Hochberg method.

### 4.6. Integrative RNA–CNA Concordance Analysis

To determine whether transcriptomic differences between YA and OI tumors were associated with structural genomic alterations, an integrative RNA–CNA concordance analysis was performed. An initial broad concordance analysis classified genes as concordant when RNA expression changes were directionally consistent with CNA patterns: upregulated genes with increased gain frequency in YA tumors, or downregulated genes with increased loss frequency in YA tumors. Genes meeting either criterion were classified as showing broad RNA–CNA concordance. To prioritize biologically meaningful associations and reduce noise from minimal CNA frequency differences, a stricter effect-size threshold was subsequently applied. Genes were classified as strict concordant only if they satisfied one of the following criteria: upregulated genes with ΔGain ≥ 0.10 and downregulated genes with ΔLoss ≥ 0.10. This threshold required a minimum 10% difference in CNA frequency between YA and OI tumors. An integration score was calculated to prioritize genes showing concordant CNAs and transcriptional changes. The absolute log_2_ fold change (|log_2_FC|) and the CNA difference (ΔGain or ΔLoss) were independently normalized using min–max scaling (0–1), and the integration score was computed as the product of the normalized values (Table 1). The top 40 genes according to the integration score were retained as the final RNA–CNA concordant gene set for downstream sensitivity analysis, external validation, functional enrichment, correlation, and network analysis.

### 4.7. External Validation Cohort

External validation of the prioritized RNA–CNA concordant gene set was performed using the independent RNA-sequencing dataset GSE229904 from the Gene Expression Omnibus (GEO) (Table 2; accessed on 6 July 2026). The GSE229904 dataset initially contained 138 records. During data curation, two duplicate sample records were identified and removed, leaving 136 unique samples. Expression and clinical metadata were then integrated, and samples lacking the information required for phenotype classification were excluded. Using the same extreme-phenotype criteria as in the discovery cohort, the final external cohort comprised 46 tumors, including 39 OI and 7 YA tumors. Genes with a count of at least 10 in ≥20% of samples were retained, and differential expression was analyzed using DESeq2 with phenotype group as the explanatory variable (design = ~group). Positive log_2_ fold-change values indicate higher expression in YA tumors, whereas negative values indicate higher expression in OI tumors.

The 40 prioritized TCGA genes were evaluated according to gene availability, directional concordance, nominal significance (*p* < 0.05), Benjamini–Hochberg-adjusted significance (FDR < 0.05), and effect size (|log_2_FC| ≥1). Gene-level log_2_ fold changes, standard errors, 95% Wald confidence intervals, Wald statistics, nominal and adjusted *p* values, and validation outcomes are provided in Supplementary Table S9. Given the limited number of YA tumors (n = 7), this analysis was considered an exploratory assessment of expression-pattern reproducibility rather than definitive validation of the gene set or its clinical predictive value. Because comparable gene-level copy-number data were unavailable, validation was restricted to the expression component and did not independently validate the CNA component or a causal RNA–CNA relationship.

### 4.8. Correlation Analysis

To evaluate exploratory associations between gene expression and clinical or genomic variables, expression values of the 40 prioritized RNA–CNA-concordant genes were evaluated against preoperative PSA levels and FGA. Spearman rank correlation analysis was used for the unadjusted analyses. Correlation coefficients (ρ) and nominal *p* values were calculated for each gene–variable pair, and *p* values were adjusted using the Benjamini–Hochberg procedure within each correlation family. For the FGA analysis, because the 40 genes were selected from the same cohort using expression and CNA patterns, the resulting correlations were considered to be post-selection and exploratory.

Because tumor purity differed between the YA and OI groups, sensitivity analyses were performed for the FGA–expression associations using linear regression models adjusted for tumor purity. These analyses were restricted to the 62 tumors with available tumor-purity measurements. Regression coefficients, standard errors, 95% confidence intervals, nominal *p* values, and Benjamini–Hochberg-adjusted *p* values were calculated for each gene. The tumor-purity-adjusted analyses were considered sensitivity analyses and were not used to redefine the original 40-gene set.

### 4.9. Functional Enrichment Analysis

Functional enrichment analysis was performed to investigate the biological functions represented by the final 40-gene RNA–CNA concordant gene set. Gene Ontology (GO) Biological Process enrichment analysis was conducted using Metascape (version 2025) with default parameters [42]. Complementary pathway enrichment analyses were performed using Enrichr [43,44], including Kyoto Encyclopedia of Genes and Genomes (KEGG) [45], Reactome [46], WikiPathways [47], and MSigDB Hallmark gene sets [48]. Enrichment significance was assessed using the adjusted *p*-values provided by each platform. Representative genes contributing to significantly enriched GO terms were extracted from the Metascape “Hits” annotation and summarized in a gene–pathway involvement matrix. Functional annotations obtained from the different enrichment platforms were compared to identify recurrent biological processes represented within the concordant gene set.

### 4.10. Protein–Protein Interaction and Network Topology Analysis

Protein–protein interaction (PPI) analysis was performed using Metascape (version 2025) [41] to investigate functional interactions among genes included in the final 40-gene RNA–CNA concordant gene set. Interaction networks were generated using experimentally validated and curated protein interactions integrated by Metascape. Functional modules were identified using the Molecular Complex Detection (MCODE) algorithm [49]. Network visualization and topological analyses were performed in Cytoscape (version 3.10.4) [50]. Network topology parameters were calculated using the NetworkAnalyzer application [51]. Hub genes were identified using the CytoHubba plugin based on the Maximal Clique Centrality (MCC) algorithm [52], and genes with the highest MCC scores were considered key network nodes for subsequent biological interpretation.

### 4.11. Statistical Analysis and Visualization

All statistical analyses were performed in R version 4.6.1 (June 2026). Data manipulation and visualization were conducted using standard R packages including DESeq2, dplyr, ggplot2 and pheatmap. Continuous variables are reported as medians or means as appropriate. All statistical tests were two-sided. Statistical significance was defined as *p* < 0.05 unless otherwise indicated. Statistical significance of enriched terms was assessed using the cumulative hypergeometric test, and *p*-values were adjusted for multiple comparisons using the Benjamini–Hochberg false discovery rate (FDR) correction. Enriched terms with an adjusted *p*-value < 0.05 were considered statistically significant. Representative GO terms and signaling pathways were ranked according to enrichment significance and used for downstream biological interpretation and visualization.

## 5. Conclusions

In this study, we performed an integrative multi-omics analysis of extreme clinical phenotypes within the TCGA-PRAD cohort to characterize molecular features associated with two predefined extreme clinical phenotypes within PCa. YA tumors exhibited higher genomic instability, increased PTEN deletion frequency, distinct molecular features, and widespread RNA–CNA-concordant transcriptional changes. Integrative RNA–CNA analysis prioritized an exploratory 40-gene set, predominantly characterized by copy-number loss-associated downregulation. Sensitivity analyses showed that 8 of the 40 genes retained both the predefined statistical and effect-size criteria after adjustment for tumor purity, whereas only RHOU retained both criteria after additional adjustment for pathological T stage and nodal status. External expression analysis provided partial directional concordance but did not yield FDR-significant associations, and the limited overlap between externally concordant and covariate-robust genes further supports the exploratory nature of the findings. Accordingly, the prioritized genes and the HCAR1–AGT–DRD2–HCAR3 network module should be considered hypothesis-generating candidates rather than validated biomarkers or evidence of CNA-driven transcriptional mechanisms. Independent cohorts, functional studies, and prospective validation will be required to determine whether these molecular features have reproducible biological or clinical relevance. Because age and tumor aggressiveness were jointly incorporated into the definition of the YA and OI groups, the observed molecular differences should not be interpreted as age-specific effects independent of tumor grade.

## Figures and Tables

**Table 1:** Top 40 prioritized genes identified by integrative analysis of gene expression and copy number alterations.

Rank	Gene	Log_2_ Fold Change (RNA-seq)	Differential Expression	Copy Number Alteration	Integration Score
1	*CDC20B*	−2.412	Down	Loss	0.451
2	*HSPB3*	−1.886	Down	Loss	0.382
3	FAM216B *	−2.789	Down	Loss	0.381
4	CARTPT *	−1.842	Down	Loss	0.373
5	*MAB21L1*	−2.070	Down	Loss	0.342
6	*TMEM132D*	−1.382	Down	Loss	0.335
7	*TMED6*	−1.750	Down	Loss	0.316
8	*HNF1A*	−2.126	Down	Loss	0.302
9	*PADI3*	−2.360	Down	Loss	0.299
10	*TMEM132C*	−1.060	Down	Loss	0.292
11	CA2 *	1.412	Up	Gain	0.277
12	*ASPA*	−1.548	Down	Loss	0.252
13	*ANKDD1B*	−1.093	Down	Loss	0.248
14	*HCAR1*	−1.337	Down	Loss	0.235
15	*MYOCD*	−1.014	Down	Loss	0.232
16	*IGSF1*	−1.630	Down	Loss	0.232
17	*CAPNS2*	−1.886	Down	Loss	0.227
18	*CRTAC1*	−1.170	Down	Loss	0.219
19	*CDH8*	−1.678	Down	Loss	0.217
20	*GALNT9*	−1.158	Down	Loss	0.214
21	*SLITRK6*	−1.171	Down	Loss	0.201
22	*HCAR3*	−1.121	Down	Loss	0.197
23	C15orf48 *	−1.737	Down	Loss	0.194
24	*CYP2C8*	−1.004	Down	Loss	0.188
25	*PLA2G2C*	−1.464	Down	Loss	0.186
26	AGT *	1.692	Up	Gain	0.184
27	*HSPB8*	−1.044	Down	Loss	0.183
28	ELMOD1 *	−1.681	Down	Loss	0.183
29	*MCF2*	−1.255	Down	Loss	0.179
30	*F7*	−1.223	Down	Loss	0.177
31	GD*F10*	−1.365	Down	Loss	0.173
32	RHOU *†	1.548	Up	Gain	0.169
33	*PLAC9*	−1.280	Down	Loss	0.165
34	*MT1M*	−1.293	Down	Loss	0.156
35	*F10*	−1.013	Down	Loss	0.147
36	*DRD2*	−1.182	Down	Loss	0.139
37	*SLC6A14*	−1.147	Down	Loss	0.135
38	*CAPN6*	−1.132	Down	Loss	0.133
39	HS3ST2 *	1.016	Up	Gain	0.120
40	*ASPM*	1.017	Up	Gain	0.111

Integration score: product of the min–max normalized absolute log_2_ fold change and CNA difference (ΔGain or ΔLoss). * Genes retaining both predefined criteria after tumor-purity adjustment (FDR-adjusted *p* < 0.05 and |log_2_FC| ≥ 1). † Gene retaining both predefined criteria after adjustment for tumor purity, pathological T stage, and nodal status.

**Table 2:** External expression analysis of the 40 prioritized genes in the independent GSE229904 cohort.

Gene	Expression Direction		Same Direction	Nominal Significance	Validation Status
	TCGA	GEO			
*PLA2G2C*	Down	Down	Yes	Yes	Concordant, nominally significant, |log_2_FC| ≥ 1
*ELMOD1*	Down	Down	Yes	Yes	Concordant, nominally significant, |log_2_FC| ≥ 1
*PLAC9*	Down	Down	Yes	Yes	Concordant, nominally significant, |log_2_FC| ≥ 1
*HSPB8*	Down	Down	Yes	Yes	Concordant, nominally significant, |log_2_FC| ≥ 1
*MAB21L1*	Down	Down	Yes	Yes	Concordant, nominally significant, |log_2_FC| ≥ 1
*RHOU*	Up	Up	Yes	Yes	Concordant, nominally significant
*MYOCD*	Down	Down	Yes	Yes	Concordant, nominally significant, |log_2_FC| ≥ 1
*MT1M*	Down	Down	Yes	Yes	Concordant, nominally significant, |log_2_FC| ≥ 1
*HSPB3*	Down	Down	Yes	Yes	Concordant, nominally significant, |log_2_FC| ≥ 1
*F10*	Down	Down	Yes	Yes	Concordant, nominally significant
*CARTPT*	Down	Up	No	Yes	Nominally significant, opposite direction
*ASPA*	Down	Down	Yes	No	Directionally concordant
*F7*	Down	Down	Yes	No	Directionally concordant
*DRD2*	Down	Down	Yes	No	Directionally concordant
*IGSF1*	Down	Down	Yes	No	Directionally concordant
*CA2*	Up	Up	Yes	No	Directionally concordant
*TMED6*	Down	Down	Yes	No	Directionally concordant
*CYP2C8*	Down	Down	Yes	No	Directionally concordant
*FAM216B*	Down	NA	NA	NA	Not available in GEO
*ASPM*	Up	Down	No	No	Opposite direction
*GDF10*	Down	Down	Yes	No	Directionally concordant
*CRTAC1*	Down	Up	No	No	Opposite direction
*CDH8*	Down	Up	No	No	Opposite direction
*TMEM132D*	Down	Down	Yes	No	Directionally concordant
*HS3ST2*	Up	Up	Yes	No	Directionally concordant
*HCAR3*	Down	Up	No	No	Opposite direction
*TMEM132C*	Down	Down	Yes	No	Directionally concordant
*PADI3*	Down	Up	No	No	Opposite direction
*MCF2*	Down	Down	Yes	No	Directionally concordant
*SLC6A14*	Down	Down	Yes	No	Directionally concordant
*SLITRK6*	Down	Down	Yes	No	Directionally concordant
*CDC20B*	Down	NA	NA	NA	Not available in GEO
*CAPNS2*	Down	Up	No	No	Opposite direction
*HCAR1*	Down	Up	No	No	Opposite direction
*CAPN6*	Down	Down	Yes	No	Directionally concordant
*AGT*	Up	Up	Yes	No	Directionally concordant
*GALNT9*	Down	Down	Yes	No	Directionally concordant
*ANKDD1B*	Down	Up	No	No	Opposite direction
*C15orf48*	Down	Up	No	No	Opposite direction
*HNF1A*	Down	NA	NA	NA	Not available in GEO

TCGA: The Cancer Genome Atlas; GEO: Gene Expression Omnibus; NA: Gene not identified in validation datasets.

## Data Availability

The datasets analyzed in this study are publicly available through The Cancer Genome Atlas Prostate Adenocarcinoma (TCGA-PRAD) project via the Genomic Data Commons (GDC) Data Portal (https://portal.gdc.cancer.gov/) and cBioPortal for Cancer Genomics (https://www.cbioportal.org/). All TCGA and GEO datasets used in this study were publicly available and were accessed and analyzed in accordance with the respective data-use policies and terms of use. The supplementary datasets, analysis files, and custom R scripts generated and/or used in this study, including clinical and genomic metadata, RNA-seq differential expression results, copy-number alteration and gene-expression data, functional enrichment results, MCODE clustering results, Cytoscape network analysis files, and scripts used for data processing and downstream analyses, are publicly available in Zenodo at https://doi.org/10.5281/zenodo.22106184.

## Supplementary Materials

The following supporting information can be downloaded at: https://www.mdpi.com/article/10.3390/ijms27188316/s1.

## Abbreviations

The following abbreviations are used in this manuscript:

AR	Androgen Receptor
AR-V7	Androgen Receptor Splice Variant 7
CNA	Copy Number Alteration
DESeq2	Differential Expression Sequencing 2
DDR	DNA Damage Repair
DEG	Differentially Expressed Gene
FDR	False Discovery Rate
FGA	Fraction Genome Altered
GEO	Gene Expression Omnibus
GO	Gene Ontology
GISTIC2	Genomic Identification of Significant Targets in Cancer 2.0
KEGG	Kyoto Encyclopedia of Genes and Genomes
MCC	Maximal Clique Centrality
MCODE	Molecular Complex Detection
MSigDB	Molecular Signatures Database
OI	Old Indolence-Associated
PCa	Prostate Cancer
PI3K	Phosphoinositide 3-Kinase
PPI	Protein–Protein Interaction
PSA	Prostate-Specific Antigen
RNA-seq	RNA Sequencing
SCNA	Somatic Copy Number Alteration
TCGA	The Cancer Genome Atlas
TCGA-PRAD	The Cancer Genome Atlas Prostate Adenocarcinoma
TP53	Tumor Protein p53
YA	Young Aggressive
